# A Novel Snow Leopard Optimization for High-Dimensional Feature Selection Problems

**DOI:** 10.3390/s24227161

**Published:** 2024-11-07

**Authors:** Jia Guo, Wenhao Ye, Dong Wang, Zhou He, Zhou Yan, Mikiko Sato, Yuji Sato

**Affiliations:** 1 Hubei Key Laboratory of Digital Finance Innovation, Hubei University of Economics, Wuhan 430205, China; guojia@hbue.edu.cn (J.G.);; 2 School of Information Engineering, Hubei University of Economics, Wuhan 430205, China; 3 Hubei Internet Finance Information Engineering Technology Research Center, Hubei University of Economics, Wuhan 430205, China; 4 Faculty of Computer and Information Sciences, Hosei Universituy, Tokyo 184-8584, Japan; 5 College of Engineering, Huazhong Agricultural University, Wuhan 430070, China; 6 College of Informatics, Huazhong Agricultural University, Wuhan 430070, China; 7 Department of Information and Telecommunication Engineering, School of Information and Telecommunication Engineering, Tokai University, Tokyo 108-8619, Japan

**Keywords:** snow leopard optimization, meta-heuristic, feature selection, high-dimensional optimization

## Abstract

To address the limitations of traditional optimization methods in achieving high accuracy in high-dimensional problems, this paper introduces the snow leopard optimization (SLO) algorithm. SLO is a novel meta-heuristic approach inspired by the territorial behaviors of snow leopards. By emulating strategies such as territory delineation, neighborhood relocation, and dispute mechanisms, SLO achieves a balance between exploration and exploitation, to navigate vast and complex search spaces. The algorithm’s performance was evaluated using the CEC2017 benchmark and high-dimensional genetic data feature selection tasks, demonstrating SLO’s competitive advantage in solving high-dimensional optimization problems. In the CEC2017 experiments, SLO ranked first in the Friedman test, outperforming several well-known algorithms, including ETBBPSO, ARBBPSO, HCOA, AVOA, WOA, SSA, and HHO. The effective application of SLO in high-dimensional genetic data feature selection further highlights its adaptability and practical utility, marking significant progress in the field of high-dimensional optimization and feature selection.

## 1. Introduction

With the advancement of technology, the data collected by sensors have become increasingly high-dimensional and large in scale. The primary challenges in processing high-dimensional sensor data are the curse of dimensionality and data redundancy. As the number of features generated by sensors skyrockets, the feature space becomes exceedingly complex, making it difficult for traditional algorithms to efficiently search and often leading to local optima. Moreover, sensor data typically contain a large number of redundant and irrelevant features, which increases computational costs and reduces model accuracy. Therefore, feature selection is crucial, as selecting the most informative features not only enhances model performance but also reduces computational complexity, improving the ability to handle high-dimensional data. The snow leopard optimization (SLO) algorithm’s main contribution to addressing the issue of feature selection in high-dimensional sensor data lies in its ability to effectively mitigate the curse of dimensionality and optimize the feature selection process. The experimental results demonstrated that SLO provides a high-precision solution for sensor data feature selection problems.

In recent years, more and more complex real-world problems can be abstracted into mathematical models. Part of these mathematical models are for optima problems in optimization problems. Scholars usually use optimization algorithms [1] to find the optimal solutions to optimization problems. In the real world, optimization algorithms can solve practical problems and improve efficiency. To deal with building energy optimization problems [2,3,4], the butterfly optimization algorithm (BOA), the pelican optimization single candidate optimizer (POSCO), and the greedy strategy-based adaptive particle swarm optimization (GAPSO) algorithm were proposed. The hybrid sine cosine algorithm (HSCA) and the refined particle swarm optimization algorithm were used to solve the engineering design optimization problems [5,6]. The wrapper feature selection technique anchored on the whale optimization algorithm (WOA) [7] was introduced to optimize feature subset identification. The enhanced lemur optimization (ELO) [8] algorithm was used to determine the most optimal relevant features. Many researchers have proposed new optimization methods to solve healthcare problems [9], image segmentation [10], and cost-effectiveness problems [11,12].

This research sought to tackle the intricate challenges of high-dimensional optimization, where the vastness and complexity of the search space often hinder the performance of traditional algorithms. The aim of developing the snow leopard optimization (SLO) algorithm was to provide a robust solution that can efficiently navigate and solve these complex problems by striking an effective balance between exploration and exploitation. Through this work, our research aspires to advance optimization techniques, especially in fields requiring high-dimensional feature selection, such as bioinformatics and environmental science, where current methods may fall short.

## 2. Related Work

Optimization algorithms are great tools for solving real-life problems, especially in the fields of healthcare, engineering design, image processing, etc., and play an important role in reducing costs, optimizing system performance, and improving accuracy. However, optimization problems, including constrained optimization problems [13], nonlinear optimization problems [14], and combinatorial optimization problems [15], have become more complex and challenging. Many scholars have proposed meta-heuristic optimization algorithms to better solve these optimization problems. Meta-heuristic algorithms [16,17,18] are a general class of optimization frameworks usually inspired by biological behavioral strategies [19,20,21,22], natural theories [23], and phenomena [24]. Abdollahzadeh [25] introduced the African vultures optimization algorithm (AVOA) to better balance diversity and resonance and improve optimization performance. Wang [26] introduced artificial rabbit optimization (ARO) inspired by rabbit survival strategies in nature. Guo [27] introduced a novel hermit crab optimization algorithm (HCOA), inspired by the distinctive behavior of hermit crabs in searching for appropriate houses to survive. The HCOA improved the robustness and accuracy of the algorithm in high-dimensional optimization work. Agushaka [28] proposed a novel population-based meta-heuristic algorithm, the gazelle optimization algorithm (GOA), which was inspired by the adaptive survival skills of gazelles in predator-rich habitats. Hashim [29] introduced the snake optimizer (SO), emulating the distinct mating behavior of snakes. Heidari [30] introduced the Harris hawks optimizer (HHO), derived from the strategic surprise pounce behavior of Harris hawks. Xue [31] introduced the sparrow search algorithm (SSA), a novel swarm optimization method inspired by the collective knowledge of sparrows and their foraging and anti-predation tactics. Dehghani [32] introduced Tasmanian devil optimization (TDO) modeled on the feeding strategies of the Tasmanian devil. Ghaedi [33] introduced the cat hunting optimization (CHO) algorithm, which adjusted the search radius around optimal solutions using trigonometric relations such as the cosine. The CHO delays rapid prey targeting, enhancing its capability to discover efficient global solutions. Hashim [34] proposed the honey badger algorithm (HBA), which was inspired by the intelligent foraging behavior of honey badgers.

Developments in fields such as oil [35,36] and environmental science [37,38] have introduced new challenges to existing technologies. In the task of feature selection [39,40], researchers have proposed numerous novel methods [41,42].

Nature-inspired optimization algorithms can solve existing optimization problems well, but these algorithms still have many limitations in solving certain optimization problems, such as easily falling into local optima [43]. To address these limitations, many researchers have proposed improved strategies based on existing optimization algorithms, to enhance their ability to solve optimization problems. These improvement methods can be divided into the following categories: adjusting parameters [44,45,46,47,48], improving the initialization strategy [49,50,51,52,53], changing the search strategy [54,55,56,57,58,59,60], and combining other optimization algorithms [61,62,63].

These optimization algorithms perform well in solving most optimization problems. However, as real-world problems become more complex, the dimensions of these optimization problems increase. Feature selection for genetic data is an example of a high-dimensional realistic optimization problem. High-dimensional genetic data have a large number of redundant or correlated features [64]. Reducing the number of features helps to improve the generalization performance of a classification model and avoid overfitting [65]. As the dimensionality of optimization problems increases, it becomes more challenging to efficiently explore the solution space. High-dimensional problems have more decision parameters and the search space becomes deeper and wider. When dealing with high-dimensional problems, it is necessary for optimization algorithms to better balance global and local search. In high-dimensional problems, local optima also occur more frequently. The performance of optimization algorithms in solving high-dimensional problems can be improved by increasing their ability to escape from local optima. When addressing high-dimensional optimization problems, it is easy for HHO to become trapped in local optima, because of its low global exploration efficiency. Due to the lack of a powerful local exploitation capacity, the horse herd optimization algorithm (HOA) [66] and SSA performed worse when solving high-dimensional optimization problems.

To face these challenge, we introduced the novel snow leopard optimization (SLO), which was inspired by the behavior of the snow leopard in nature, to better solve high-dimensional problems. More specifically, the main research contributions of this paper are as follows:

- A novel snow leopard optimization (SLO) algorithm is proposed, which has a better performance on high-dimensional problems.
- The four different strategies of the SLO can provide a better balance between global search and local search.
- Compared with eight well-known optimizers, the HSO was verified to be more effective in solving high-dimensional problems, including the CEC2017 benchmark and feature selection of high-dimensional genetic data.

The structure of the remaining sections of the manuscript is as follows: Section 2 provides a detailed description of the algorithm we propose. Section 3 details the simulation experiments and their outcomes. Finally, Section 4 concludes the paper and outlines potential future research directions.

## 3. Methods

High-dimensional problems are caused by large increases in decision variables. When increasing the decision variables, the solution space becomes wider and the number of local optima increases. Balancing the global and local search of a solution space and increasing the ability of escape from local optima are the main challenges of high-dimensional problems. To better solve these problems, we developed a novel snow leopard optimization (SLO), which was inspired by the real-world behavior of snow leopards, including territorial delineation, territory encroachment, and resource pillage. In this section, we discuss some details of the snow leopard and the SLO.

### 3.1. Behavior of Snow Leopards

The snow leopard is a big cat, known as the “monarch of the icy realms”. They are often found in icy alpine bare rock and cold desert zone environments. Snow leopards live in both solitary habitats, where they continuously attempt to seek a mate, and group habitats, mainly during the early stages of mating and cub rearing. Snow leopards typically have extensive territories, and the size of this territory depends on environmental conditions and the availability of food resources. The range of territory may vary from tens of square kilometers to several hundreds of square kilometers. This territory provides food, habitat, and resources for reproduction. Snow leopard territories are also gender-related. During the mating season, males and females jointly seek food, and this cooperative approach helps females acquire sufficient nutrition to support pregnancy. Snow leopards without mating partners attempt to move in the vicinity of those without mates, trying to seize opportunities for mating. They generally operate within their territorial boundaries. However, in the winter, as temperatures drop, they compete for food outside their territorial range, and this behavior becomes more frequent as temperatures decline.

### 3.2. Snow Leopard Optimization

**Figure 1:**
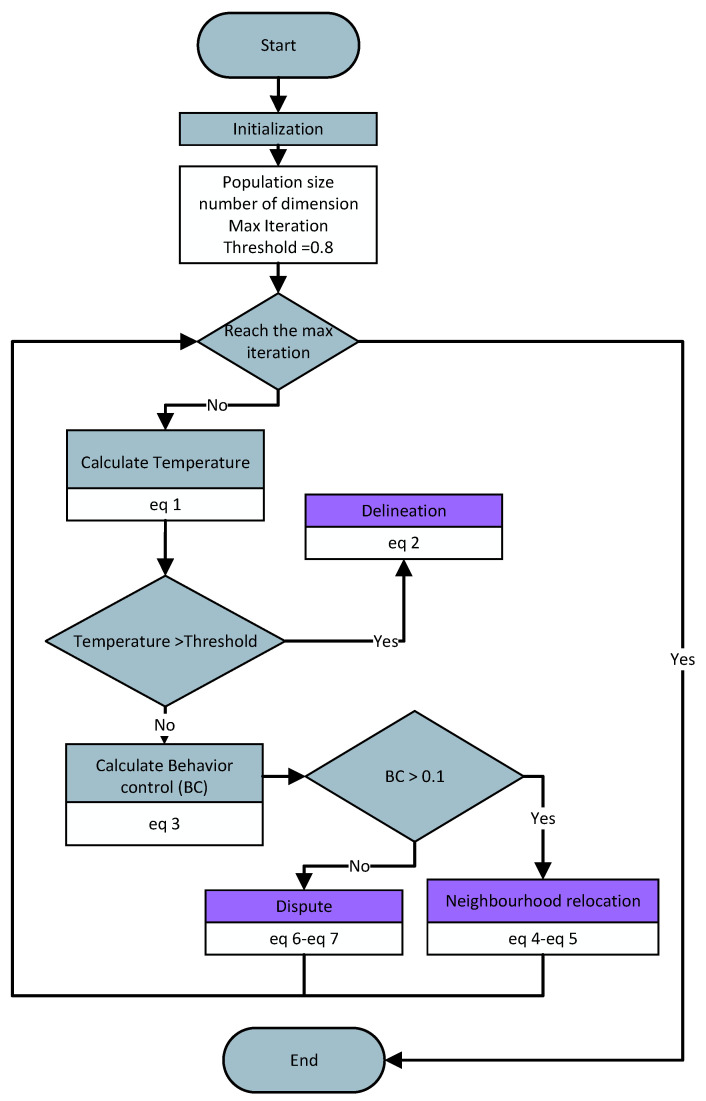
Flowchart of SLO.

Inspired by snow leopard behavior, we introduced a novel meta-heuristic optimization algorithm, snow leopard optimization (SLO). The process of snow leopards seeking territories can be abstracted as an optimization algorithm searching for the optimal solution. In SLO, the search space represents the habitat of snow leopards in the natural world. The territories of individuals in the search space correspond to a solution. Each snow leopard has territorial superiority, which can be associated with the fitness of the optimization function. Territorial superiority involves many factors such as resource abundance, geographical position, and food availability. These influencing factors affect territorial superiority just like decision variables impact an optimization problem. Snow leopards with better territories have a better territorial superiority fitness. Among all individuals, the snow leopard with the best territorial superiority is referred to as the “Leopard King”. In the natural world, the territorial behaviors of snow leopards are often associated with gender, territorial superiority, and living arrangements. Based on the living arrangements and gender of snow leopards, we categorize them into solitary males, solitary females, co-residing males, and co-residing females. In the initialization phase, each male snow leopard is randomly assigned as either solitary or co-residing. Co-residing male snow leopards are assigned a cohabiting female snow leopard as an mating object. Different types of snow leopards exhibit different territorial behaviors. Furthermore, temperature also influences the behavior of snow leopards. In the SLO, we introduce the concept of temperature, which is related to the number of iterations. The equation for the temperature is shown in Equation (1). When the temperature is high, snow leopards delineate territory. However, neighborhood relocation and dispute only occur when the temperature is low. As the temperature decreases, the probability of resource disputes increases, while the proportion of neighborhood relocations decreases. These strategies were used to guide the optimization algorithm to improve its efficiency and performance. A flowchart of SLO is shown in Figure 1.

(1) $$Temperature=1-\frac{2*t}{t+MAX\_T}$$

where *t* and MAX_T are the number of current and the max iterations, respectively.

### 3.3. Delineation

The behavior of snow leopard territorial delineation is a crucial behavior used to ensure the availability of survival resources. In SLO, delineation takes place in the early iterations, when the Temperature is above a threshold (threshold = 0.8). Snow leopards search for territories near members of the opposite sex with the same living arrangement. Solitary snow leopards can secure more mating opportunities, while co-residing snow leopards can acquire territories with better access to survival resources. The delineation of these territories is not random. In SLO, the territorial delineation of the snow leopards is based on the superiority of their territory and the superiority of the selected opposite-sex territory. Stronger individual snow leopards tend to have better territorial superiority. When choosing targets, snow leopards avoid those with a large gap in superiority, in order to minimize competition. As a result, snow leopards with poorer territorial superiority do not approach snow leopards with better territorial superiority, but rather stay away from them.The calculation of candidate territorial delineation for snow leopards is as shown in Equation (2). With the help of this strategy, individuals in SLO can continuously achieve a better territorial delineation. This measure prevents the population from converging too quickly in the early stages and increases the ability for global search.

(2) $$CandiT\left(Type\left(i\right)\right)=T\left(Co_{id}\right)\pm c_{1}*\frac{\left((Up-Low)*rand+Low\right)}{{\left(\frac{fitness(T\left(Co_{id}\right))}{fitness(T(Type(i)))}+1\right)}^{2}}$$

where fitness() is a function to calculate territorial superiority. T(Type(i)) and $T(Co_{id})$ represent the territory of the *i*-th snow leopard, with different types that include solitary males, solitary females, co-residing males, and co-residing females, and the territory of the same-type counterparts with a different gender. Up and Low are the maximum and minimum values of the search space range. rand indicates a random value from 0 to 1. $c_{1}$ is a constant with a value of 0.5.

### 3.4. Neighborhood Relocation

The neighborhood relocation behavior of the snow leopard describes the situation in which they leave their existing territory to seek a better one and relocate to a new territory nearby. Neighborhood relocation is a strategy for snow leopards to adapt to changes and ensure their survival. However, it also came with various challenges, including competition with other snow leopards, the difficulty of finding a new territory, and the advantages of the new territory. Due to variations in their competitors, different types of snow leopards have different relocation methods. Furthermore, the occurrence of territorial neighborhood relocation is related to temperature. The lower the temperature, the more likely snow leopards are to change their territories. Therefore, as the temperature decreases, territorial encroachment occurs more frequently. In SLO, the behavioral control (BC) formula is designed to adaptively control the frequency of occurrence of territorial neighborhood relocation. The calculation method for BC is shown in Equation (3).

(3) BC=rand∗(1−Temperature)

where rand is a random value from 0 to 1. The Temperature can be calculated using Equation (1).

Neighborhood relocation of territory only occurs when BC is more than 0.1. In this strategy, the snow leopards select co-residing snow leopards of the same gender as their encroachment targets. These snow leopards undergo repeated territorial shifts, which are influenced by their competitive objectives. Furthermore, the relocations of male snow leopards are impacted by the leopard king, which has the best territory in the area. The calculation formula for generating candidate territories during territorial neighborhood relocation of snow leopards is shown in Equations (4) and (5).

(4) $$CandiT\left(Type\left(i\right)\right)=T\left(Type\left(i\right)\right)\pm c_{1}*BC*randn*\left(T\left(Type\left(i\right)\right)-T\left(Co_{id}\right)\right)$$

where T(Type(i)) and $T(Co_{id})$ represent the territory of the *i*-th snow leopard with different types, which includes solitary males, solitary females, co-residing males, and co-residing females, and the territory of their same-type counterparts with the same gender. randn is a function value that is generated randomly from a Gaussian distribution with an expected value of 0 and a variance of 1. $c_{1}$ is a constant with a value of 0.5.

(5) $$CandiT\left(Type\left(i\right)\right)=T\left(Type\left(i\right)\right)\pm c_{1}*BC*randn*\left(T\left(Type\left(i\right)\right)-TKing\right)$$

where T(Type(i)) and TKing represent the territory of the *i*-th snow leopard, with different types that include solitary males and co-residing males, and the territory of leopard king. randn is a function value that is generated randomly from a Gaussian distribution with an expected value of 0 and a variance of 1. $c_{1}$ is a constant with a value of 0.5.

To better explain the process of territorial neighborhood relocation in SLO, the pseudocode for the territorial neighborhood relocation strategy is shown in Algorithm 1. This strategy involves individuals continuously searching for superior territories around them, while being influenced by various factors. This enhances each individual’s local search capabilities, and at the same time, the population moves in the direction of the optimal solution, ensuring that the entire population evolves toward the optimal outcome.
**Algorithm 1** Neighborhood relocation1:Input parameters:2:Territory, $T=T_{1},T_{2},...,T_{n}$.3:King leopard territory, TKing.4:Temperature, Temperature.5:behavioral control, BC.6:Type of the snow leopard, Type.7:Number of one type snow leopard, $N_{Type}$.8:Number of Max iteration, MAX_T.9:Number of current iteration, *t*.10:$Temperature=1-2*\frac{t}{t+MAX_{T}}$.11:BC=rand∗(1−Temperature).12:Main algorithm:13:**if** rand∗BC<0.4 **then**14:    Define Type.15:    **while** $i<N_{Type}$ **do**16:        $Co_{id}$ is a random index from co-residing male or solitary male.17:        **if** Type==solitarmale||co−residingmale **then**18:           **if** rand<0.5 **then**19:               Generate CandidateTerritory by Equation (4).20:           **else**21:               Generate CandidateTerritory by Equation (5).22:           **end if**23:        **else**24:           Generate CandidateTerritory by Equation (4).25:        **end if**26:        i=i+127:    **end while**28:**end if**29:Output: Territory

### 3.5. Dispute

In the snow leopard swarm, territorial disputes of snow leopards are a natural behavior that helps them ensure an adequate supply of food and habitat resources for survival. Territorial disputes mainly occur near the leopard king and become more frequent as temperatures decrease. On the one hand, the leopard king possesses the superior territory, and on the other hand, as temperatures drop, the available survival resources within the territories sharply decrease. Superior territory provides access to greater survival resources. In SLO, both disputes and neighborhood relocations of a territory are observed when the Temperature falls below 0.8. Specially, disputes happen when BC is less than 0.1, and the likelihood increases with the number of iterations. In the behavior of disputes, the primary focus is the territory of the leopard king. Nevertheless, solitary snow leopards engage in territorial battles with individuals of the same sex to secure mating rights. The formulation for generating potential territories during disputes among snow leopards is illustrated in Equations (6) and (7).

(6) $$CandiT\left(Type\left(i\right)\right)=TKing\pm c_{1}*randn*\left(\left(1-Temperature\right)*TKing-T\left(Type\left(i\right)\right)\right)$$

where T(Type(i)) and TKing represent the territory of the *i*-th snow leopard with different types, including all kinds of the snow leopards, and the leopard king territory, respectively. randn is a function value that is generated randomly from a Gaussian distribution with an expected value of 0 and a variance of 1. $c_{1}$ is a constant with a value of 0.05. Temperature is a variable that varies with the number of iterations, and its calculation method is outlined in Equation (1).

(7) $$CandiT\left(Type\left(i\right)\right)=TKing\pm c_{1}*randn*\frac{\left(\left(1-Temperature\right)*TKing-T\left(Co_{id}\right)\right)}{\left(\frac{fitness(T(Type(i)))}{fitness(T\left(Co_{id}\right))}+1\right)}$$

where $T(Type_{id})$ and $T(Co_{id})$ represent the territory of the *i*-th snow leopard with different types, which includes solitary males, solitary females, and the territory of their same-type counterparts with the same gender. TKing is the territory of the leopard King. randn is a function value that is generated random from a Gaussian distribution with an expected value of 0 and a variance of 1. $c_{1}$ is a constant with a value of 0.05. Temperature is a variable that varies with the number of iterations, and its calculation method is outlined in Equation (1).

To provide a clear representation of how the territorial dispute behavior operates, its pseudocode is presented in Algorithm 2. With this strategy in place, individuals gravitate toward territories with global superiority. Different types of individuals are influenced by various contesting parties, resulting in diverse approaches to territorial disputes. This enhances the individual local search capabilities, ultimately improving the precision of later solutions.
**Algorithm 2** Dispute1:Input parameters:2:Territory, $T=T_{1},T_{2},...,T_{n}$.3:King leopard territory, TKing.4:Temperature, Temperature.5:behavioral control, BC.6:Type of the snow leopard, Type.7:Number of one type snow leopard, $N_{Type}$.8:Number of Max iteration, MAX_T.9:Number of current iteration, *t*.10:Main algorithm:11:$Temperature=1-2*\frac{t}{t+MAX_{T}}$.12:BC=rand∗(1−Temperature).13:**if** rand∗BC>0.4 **then**14:    Define Type.15:    **while** $i<N_{Type}$ **do**16:        $Co_{id}$ is a random index from solitary male or solitary female.17:        **if** Type=solitarymale||solitaryfemale **then**18:           **if** rand<0.5 **then**19:               Generate CandidateTerritory by Equation (6).20:           **else**21:               Generate CandidateTerritory by Equation (7).22:           **end if**23:        **else**24:           Generate CandidateTerritory by Equation (6).25:        **end if**26:        i=i+127:    **end while**28:**end if**29:Output: Territory

## 4. Experiments and Results

In this article, 7 algorithms, ETBBPSO, ARBBPSO, HCOA, AVOA, WOA, SSA, and HHO, were selected as a control group to compare with SLO. To validate the performance of SLO in high-dimensional optimization problems, the highest recommended dimension in CEC2017 was chosen as a benchmark. Eight real high-dimensional gene datasets were selected to validate the ability for feature selection. In this section, the details of these two group experiments and results are given. In the experiment, MATLAB software version 2020b on the Windows 10 operating system was used.

### 4.1. Benchmark Test

The CEC2017 is a well-know optimization problem test set. It includes a total of 29 benchmark functions, which comprise unimodal functions (F1–F2), simple multimodal functions (F3–F9), hybrid functions (F10–F19), and composition functions (F20–F29). There are 10, 30, 50, and 100 dimensions in CEC2017. The 100-dimension version was selected to conduct this test. In order to reduce the randomness of the experimental results, both SLO and the control group algorithms were subjected to 36 independent experiments. We employed the mean error (ME) to evaluate the performance of all algorithms on CEC2017. The formula for calculating the ME is shown in Equation (8). To ensure the fairness of the comparison, all algorithms used the parameters set by the original paper. All experiments were had the same population of 100, max iteration number of 10,000, and dimensions of 100.

(8) $$ME=\frac{\sum_{i=1}^{N}abs\left(Actual\;Optimum-Theoretical\;Optimum\right)}{N}$$

where *N* is the number of independent experiments. abs() represents a function used to calculate the absolute value of data. ActualOptimum and TheoreticalOptimum are the actual optimal value calculated by the algorithms and the theoretical optimal value of the test function itself.

**Figure 2:**
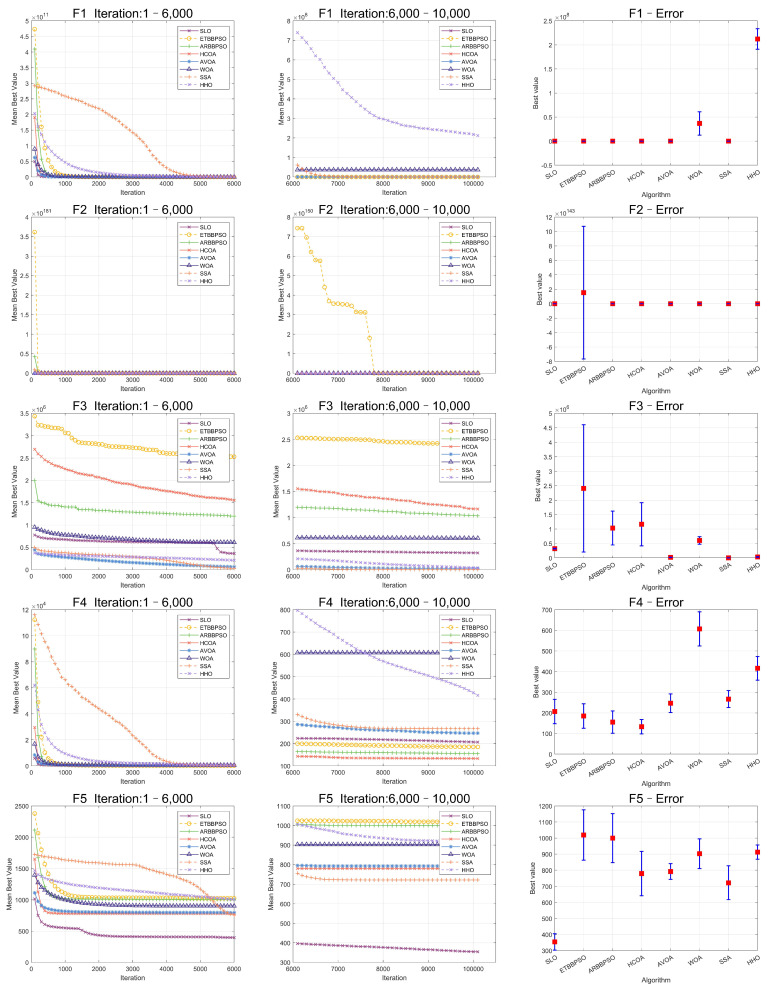
Convergence curves and error bars of SLO and control group algorithms on CEC2017 function 1–function 5.

**Figure 3:**
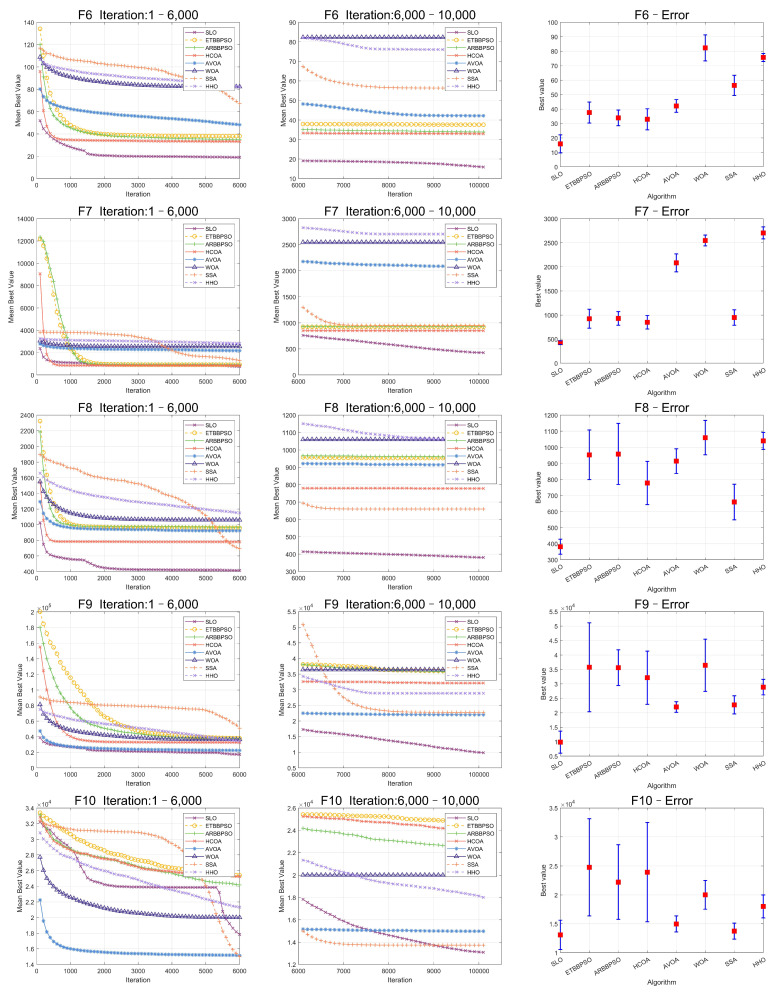
Convergence curves and error bars of SLO and control group algorithms on CEC2017 function 6–function 10.

**Figure 4:**
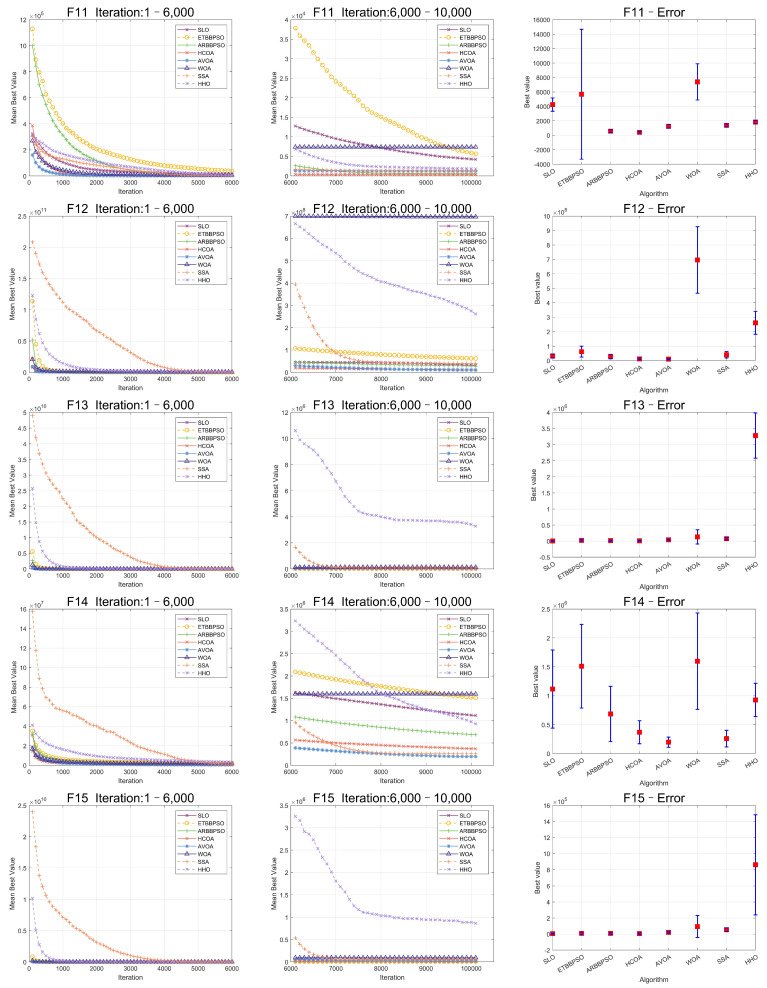
Convergence curves and error bars of SLO and control group algorithms on CEC2017 function 11–function 15.

**Figure 5:**
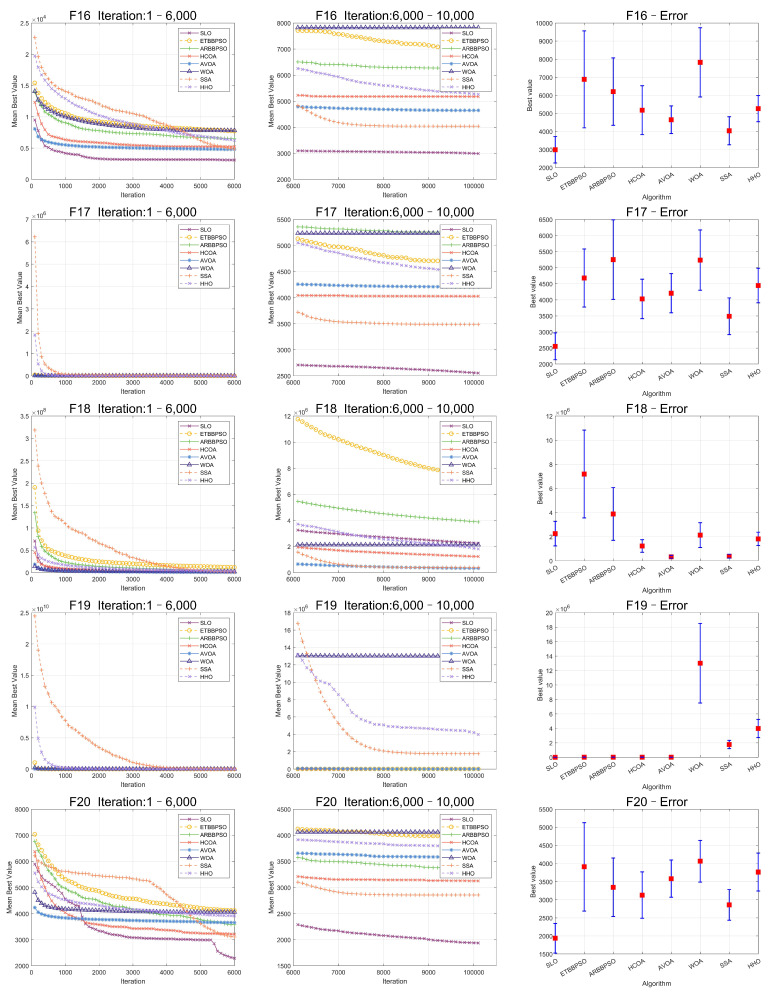
Convergence curves and error bars of SLO and control group algorithms on CEC2017 function 16–function 20.

**Figure 6:**
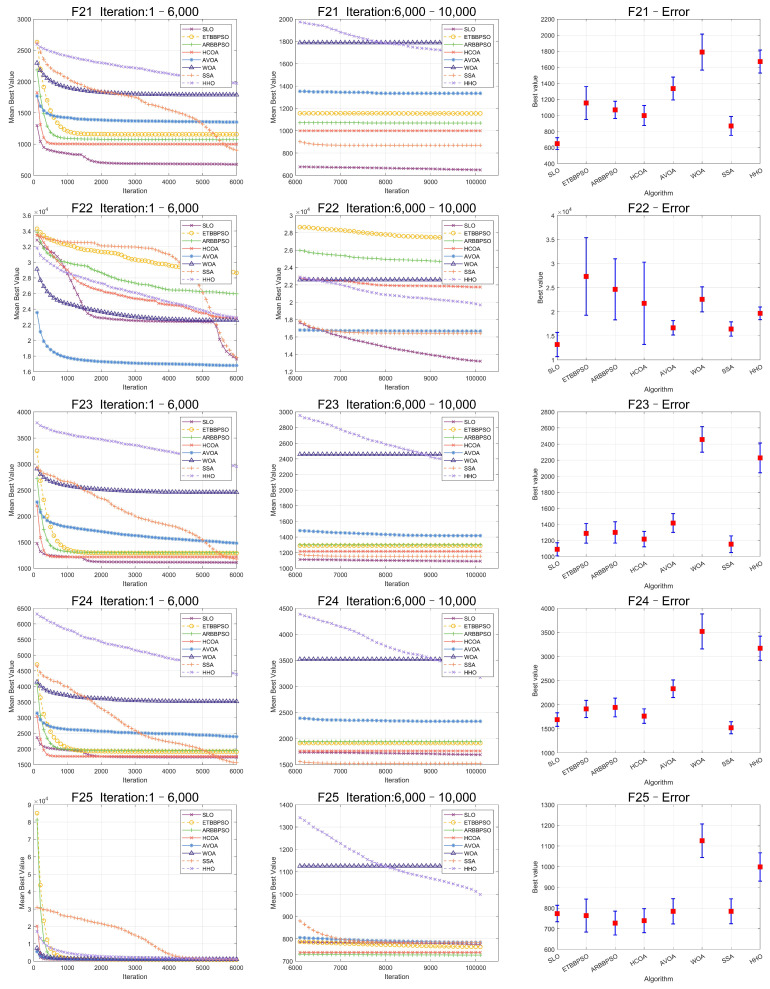
Convergence curves and error bars of SLO and control group algorithms on CEC2017 function 21–function 25.

**Figure 7:**
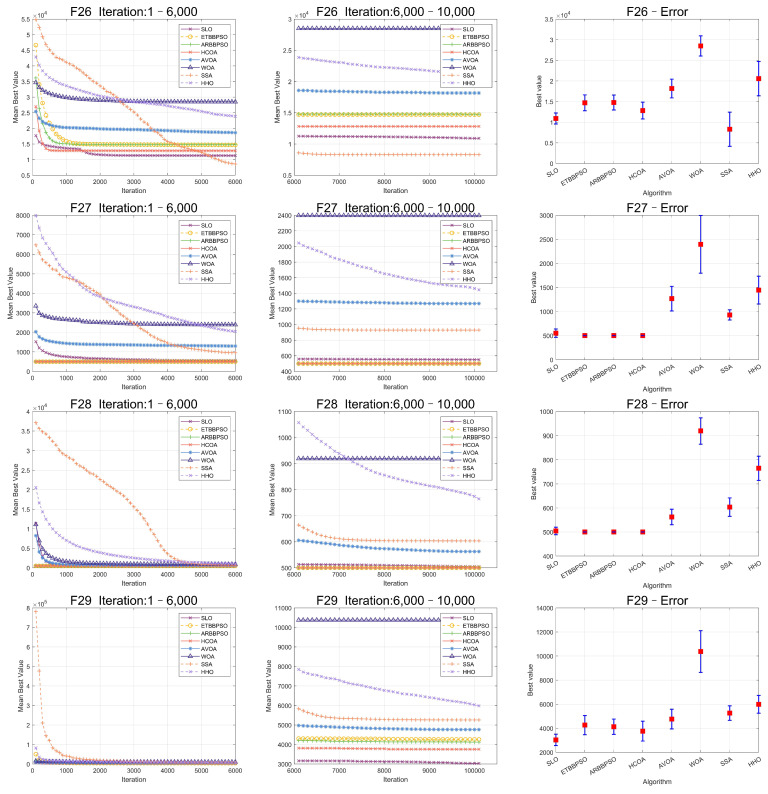
Convergence curves and error bars of SLO and control group algorithms on CEC2017 function 26–function 29.

The Friedman test was used to analyze the ME. The specific ME, standard deviation(Std), and rank are presented in Table 1 and Table 2, and the average rank of the algorithms is shown at the bottom of Table 2. To provide a more intuitive representation of the convergence process and errors for SLO and control group algorithms, the convergence process of ME for all algorithms on CEC2017, as well as the final ME error bars, are shown in Figure 2, Figure 3, Figure 4, Figure 5, Figure 6 and Figure 7.

Specifically, SLO ranked first on average among the 8 algorithms, with a score of 2.4138, which was 21.35% better than the second-ranked algorithm, HCOA. For the four different types of test functions of CEC2017, SLO obtained better optimal solutions than the control group algorithms in most of the functions with simple multimodal functions, hybrid functions, and composition functions. SLO achieved 5 first ranks compared with the control group for the 7 simple multimodal functions. SLO outperformed the other algorithms for the hybrid and composition functions, with a 60% and 50% share of the first rank. However, SLO was not as good as the other controls in solving single-peaked functions. SLO had no worst performance rankings among all 29 functions. On balance, SLO was superior in solving high-dimensional benchmark functions.

As seen in Figure 2, Figure 3, Figure 4, Figure 5, Figure 6 and Figure 7, SLO had a significant advantage in convergence speed on F5, F6, F8, F16, and F21. This was due to its territorial division strategy. Except for F10, F11, F14, and F22, SLO found the exact solution in the later stage. Meanwhile, as seen from the error bar chart, SLO had a strong robustness, with small errors in 36 independent experiments. This indicated that SLO can provide a good balance between global and local searching.

### 4.2. Feature Selection

Eight real gene datasets were selected to further validate the performance of SLO in high-dimensional problems. The details of these datasets are shown in the Table 3.

The data in the dataset were divided into a training set and test set in the ratio of 80% and 20%. The K nearest neighbor (KNN) algorithm was used as a classifier to validate the results of the feature selection. Due to the small number of training samples in the high-dimensional gene dataset, we used K-fold cross-validation to reduce the chance of error in training. The search range of all algorithms was [0,1]. The selected features [40] were determined by Equation (9).

(9) $$\left\{\begin{array}{c} X(i)<0.5\;\mathrm{Feature}\;\mathrm{is}\;\mathrm{not}\;\mathrm{selected}\\ X(i)\geq 0.5\;\mathrm{Feature}\;\mathrm{is}\;\mathrm{selected} \end{array}\right.$$

where X(i) is the value of the *i*-th dimension in the optimal individual.

The parameters used in training are shown in Table 4. Using a 10-fold cross-validation provided a reliable assessment of the model’s performance by ensuring that each data point was used for both training and validation across the different iterations [42]. In the KNN model, the value of K was set to 5 to achieve an optimal balance between generalization capability and classification accuracy [41]. In order to comprehensively measure the performance of the algorithms in feature selection, we divided the fitness of the optimization algorithms into two parts, one part was the accuracy of the classification after feature selection, and the other part was the ratio of the number of selected features to the number of original features. The fitness of feature selection was calculated as shown in Equation (10).

(10) $$fitness=\alpha *\left(1-Acc\right)+\beta *\frac{n}{N}$$

where Acc is the accuracy of classification after feature selection. *n* and *N* represent the number of features after feature selection and the total number of features, respectively. α and β are equal to 0.99 and 0.01 constants, respectively.

To reduce the variation in the experimental results, all algorithms were subjected to 20 separate experiments on each dataset. The average experimental results were chosen to compare the performance of the algorithms. The Friedman test method was used to evaluate the performance of SLO in feature selection. The results of the fitness and accuracy calculated by SLO and the control group algorithms are shown in Table 5 and Table 6. The mean number and standard deviation of the selected features are shown in Table 7.

As seen in Table 5, the fitness value of the SLO had a distinct superiority over the other algorithms. It achieved five first ranks among the eight datasets and achieved first place according to the Friedman test. SLO had the best overall rank of 1.625, which was 0.375 higher than the second ranking algorithm, AVOA.

As seen in Table 6, SLO achieved classification accuracies of over 70% for all eight datasets after feature selection. The highest classification accuracy of 98.90% was achieved on the Lymphoma dataset. In the Friedman test, SLO obtained five first and best average rankings of 1.625, which was 0.15 higher than the second ranked algorithm, AVOA.

Table 7 shows that SLO also achieved a great improvement in the number of features selected. Compared to the other algorithms, it had a minimum of six features selected in the eight datasets. SLO had the greatest advantage in the mean number of features selected on the Colon dataset, with 27.28 after selection, which accounted for 1.36% of the total features, a reduction of 1972.72 features.

**Figure 8:**
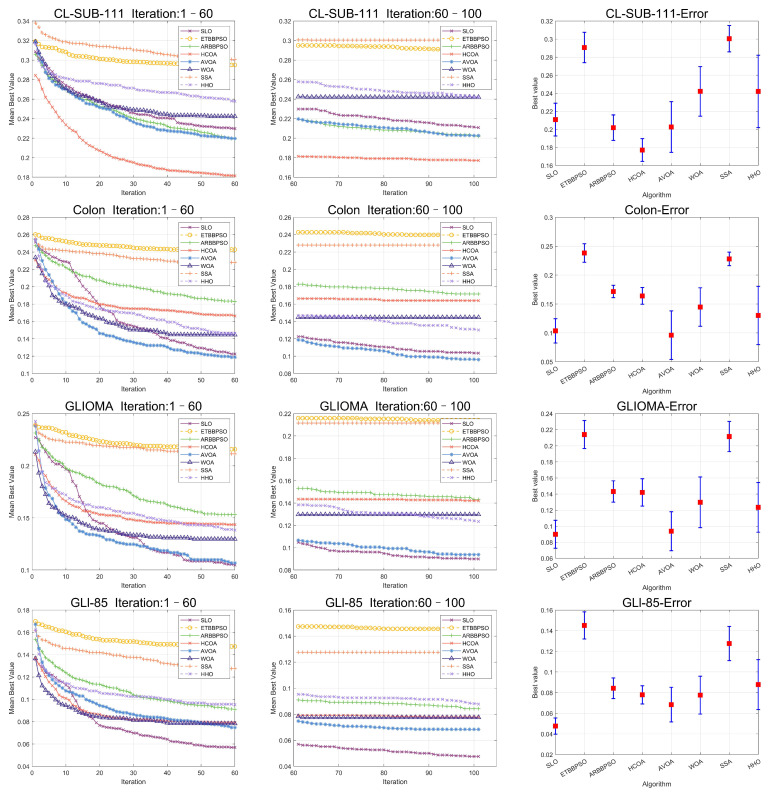
Convergence curves and error bars of SLO and the control group algorithms on CL-SUB-111, Colon, GLIOMA, and GLl-85.

**Figure 9:**
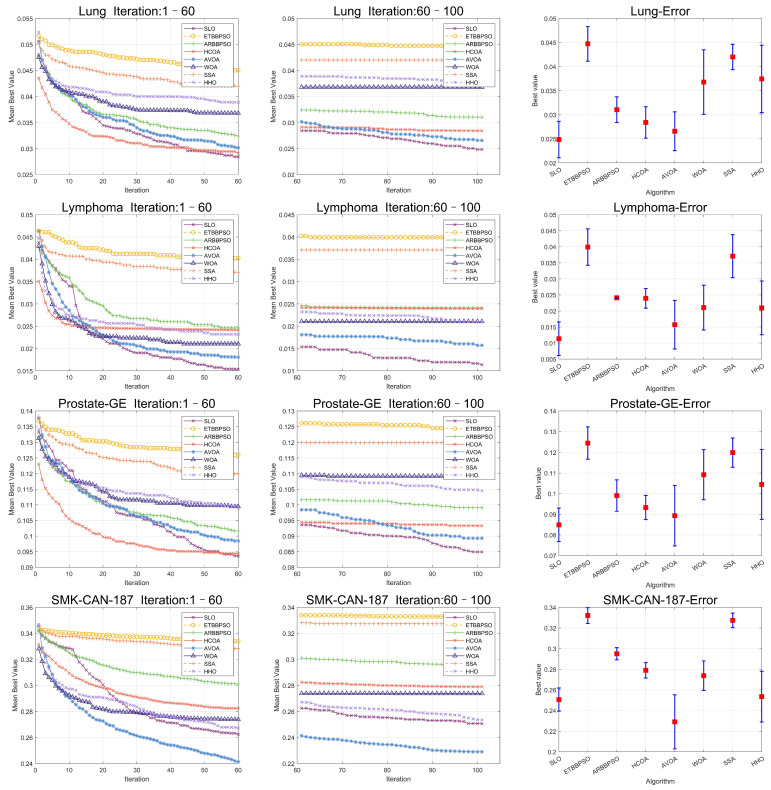
Convergence curves and error bars of SLO and the control group algorithms on Lung, Lymphoma, Prostate-GE, and SMK-CAN-187.

To clearly visualize the convergence curves of the fitness of all the algorithms on the eight datasets, as well as their error, convergence plots of the SLO and control group algorithms, as well as error bars, are shown in Figure 8 and Figure 9.

**Figure 10:**
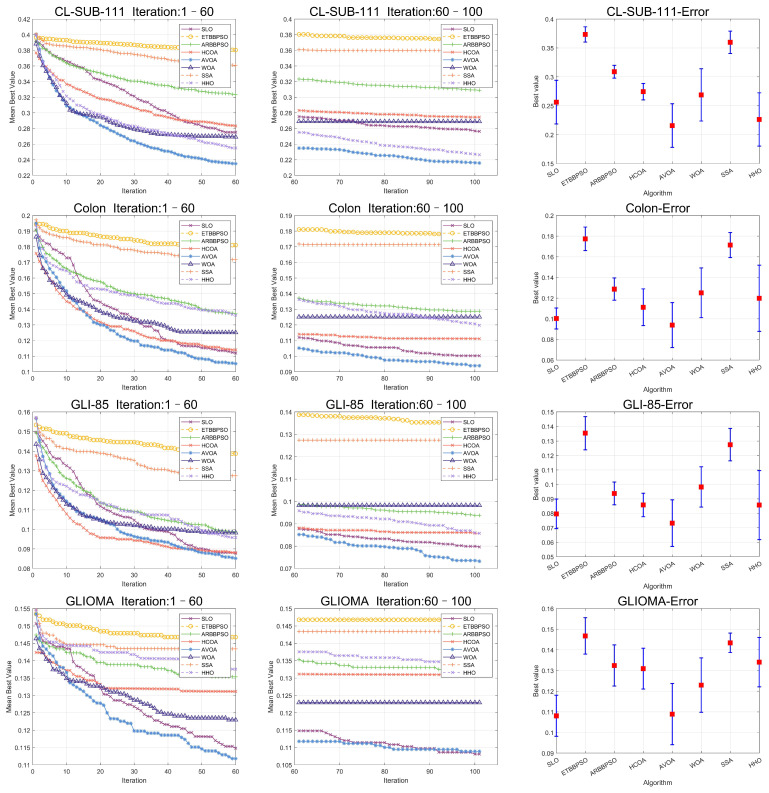
Convergence curves and error bars of SLO and the control group algorithms on CL-SUB-111, Colon, GLIOMA, and GLl-85.

**Figure 11:**
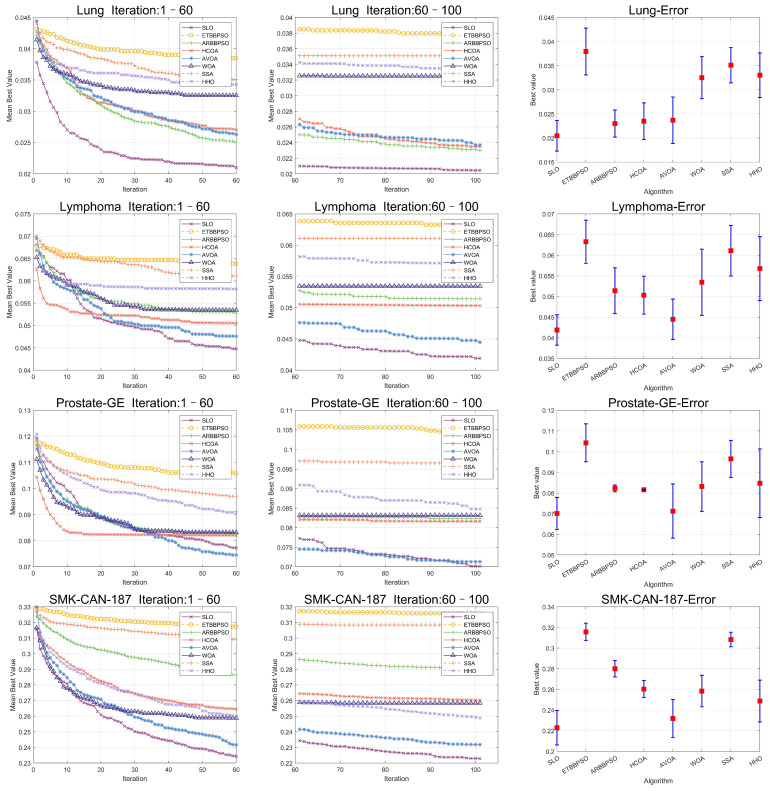
Convergence curves and error bars of SLO and the control group algorithms on Lung, Lymphoma, Prostate-GE, and SMK-CAN-187.

In addition, to enhance the persuasiveness of the experiment, 90 percent of the data were used for training and 10 percent of the data were used for testing, and the results of the experiment are shown in Table 8, Table 9 and Table 10, and the convergence plots of the SLO and control group algorithms, as well as the error bars, are shown in Figure 10 and Figure 11.

### 4.3. Discussion

From Figure 8 and Figure 9, it is evident that, while the early convergence speed of the snow leopard optimization (SLO) algorithm in the eight high-dimensional gene feature selection problems was slower than that of the other algorithms, the accuracy of its solutions was notably higher than that of the control group. This behavior can be attributed to the nature of the feature selection problem, which has a limited search range. The territory delineation strategy in SLO, while not achieving rapid convergence initially, helped prevent premature convergence by reducing the likelihood of becoming trapped in a local optimum in the early stages.

Additionally, the territorial neighborhood relocation and dispute strategies in SLO enhanced the local search efficiency, thereby improving the accuracy of the later solutions. By using these local search methods, the algorithm refined its search around promising areas in the later stages, yielding more accurate solutions.

In the comparative evaluations, SLO was benchmarked against seven well-known optimization algorithms—HCOA, ARBBPSO, ETBBPSO, AVOA, SSA, HHO, and WOA—which served as a control group. In the CEC2017 benchmark test function set, SLO achieved the highest average rank. Furthermore, when applied to eight real gene datasets, SLO outperformed the control algorithms in terms of predictive accuracy and achieved the highest average ranking for the number of selected features. This demonstrated SLO’s superior performance in maintaining accuracy, while effectively managing the challenges of high-dimensional feature selection.

## 5. Conclusions

In this paper, we introduced a novel optimization algorithm, named snow leopard optimization(SLO). SLO is a meta-heuristic algorithm inspired by the behavior of snow leopards. It primarily employs the territorial behaviors of snow leopard delineation, neighborhood relocation, and disputes to address high-dimensional optimization problems. To demonstrate the performance of SLO on high-dimensional optimization problems, we selected two types of tests, including benchmark functions and real-world problems. In the CEC2017 benchmark test function set, SLO ranked first on average. Moreover, after feature selection on eight real gene datasets, its predictive accuracy and the average statistical ranking of the number of feature selections also ranked first.

Moving forward, future work will focus on extending the SLO algorithm to tackle a wider range of optimization problems, including multi-objective and dynamic optimization challenges. Additionally, improvements in computational efficiency and adaptability will be explored, allowing SLO to be applied to real-time or resource-constrained environments.

However, some limitations of this work should be acknowledged. While SLO demonstrated strong performance on benchmark datasets, its effectiveness in extremely high-dimensional problems may decrease as the complexity of the search space increases. Moreover, like many meta-heuristic methods, the algorithm’s performance can be sensitive to parameter tuning. Future studies could explore adaptive parameter settings and hybrid techniques to further enhance the robustness and flexibility of SLO.

## Figures and Tables

**Table 1:** Experimental results of SLO, HCOA, ARBBPSO, ETBBPSO, AVOA, SSA, HHO, and WOA for F1–F15. Mean, Std, and Rand are dimensionless.

F	Data Type	SLO	HCOA	ARBBPSO	ETBBPSO	AVOA	SSA	HHO	WOA
F1	Mean	1.029 × 10^4^	1.920 × 10^4^	1.200 × 10^4^	2.239 × 10^4^	7.664 × 10^3^	7.889 × 10^3^	2.123 × 10^8^	3.686 × 10^7^
	Std	1.793 × 10^4^	2.164 × 10^4^	1.678 × 10^4^	2.344 × 10^4^	1.143 × 10^4^	1.247 × 10^4^	2.133 × 10^7^	2.429 × 10^7^
	Rank	3	5	4	6	1	2	8	7
F2	Mean	2.935 × 10^77^	3.022 × 10^76^	2.129 × 10^131^	1.531 × 10^143^	8.337 × 10^29^	1.929 × 10^38^	7.215 × 10^69^	6.394 × 10^131^
	Std	1.761 × 10^78^	1.334 × 10^77^	8.903 × 10^131^	9.184 × 10^143^	4.742 × 10^30^	1.096 × 10^39^	3.553 × 10^70^	2.597 × 10^132^
	Rank	5	4	6	8	1	2	3	7
F3	Mean	3.215 × 10^5^	1.166 × 10^6^	1.035 × 10^6^	2.405 × 10^6^	1.937 × 10^4^	4.292 × 10^3^	3.360 × 10^4^	6.047 × 10^5^
	Std	1.027 × 10^4^	7.488 × 10^5^	5.852 × 10^5^	2.199 × 10^6^	4.565 × 10^3^	2.035 × 10^3^	8.377 × 10^3^	1.298 × 10^5^
	Rank	4	7	6	8	2	1	3	5
F4	Mean	2.066 × 10^2^	1.330 × 10^2^	1.553 × 10^2^	1.851 × 10^2^	2.468 × 10^2^	2.670 × 10^2^	4.161 × 10^2^	6.072 × 10^2^
	Std	5.869 × 10^1^	3.452 × 10^1^	5.393 × 10^1^	5.944 × 10^1^	4.539 × 10^1^	4.114 × 10^1^	5.739 × 10^1^	8.273 × 10^1^
	Rank	4	1	2	3	5	6	7	8
F5	Mean	3.541 × 10^2^	7.794 × 10^2^	9.997 × 10^2^	1.019 × 10^3^	7.924 × 10^2^	7.223 × 10^2^	9.126 × 10^2^	9.027 × 10^2^
	Std	5.097 × 10^1^	1.374 × 10^2^	1.532 × 10^2^	1.568 × 10^2^	4.827 × 10^1^	1.051 × 10^2^	4.459 × 10^1^	9.233 × 10^1^
	Rank	1	3	7	8	4	2	6	5
F6	Mean	1.594 × 10^1^	3.293 × 10^1^	3.388 × 10^1^	3.759 × 10^1^	4.213 × 10^1^	5.636 × 10^1^	7.570 × 10^1^	8.232 × 10^1^
	Std	6.258	7.279	5.411	7.261	4.409	6.956	2.778	8.997
	Rank	1	2	3	4	5	6	7	8
F7	Mean	4.275 × 10^2^	8.501 × 10^2^	9.301 × 10^2^	9.239 × 10^2^	2.083 × 10^3^	9.488 × 10^2^	2.705 × 10^3^	2.548 × 10^3^
	Std	3.969 × 10^1^	1.402 × 10^2^	1.424 × 10^2^	1.970 × 10^2^	1.866 × 10^2^	1.606 × 10^2^	1.246 × 10^2^	1.131 × 10^2^
	Rank	1	2	4	3	6	5	8	7
F8	Mean	3.812 × 10^2^	7.783 × 10^2^	9.586 × 10^2^	9.533 × 10^2^	9.144 × 10^2^	6.598 × 10^2^	1.040 × 10^3^	1.061 × 10^3^
	Std	4.662 × 10^1^	1.346 × 10^2^	1.897 × 10^2^	1.538 × 10^2^	7.705 × 10^1^	1.107 × 10^2^	5.255 × 10^1^	1.062 × 10^2^
	Rank	1	3	6	5	4	2	7	8
F9	Mean	9.845 × 10^3^	3.215 × 10^4^	3.562 × 10^4^	3.576 × 10^4^	2.201 × 10^4^	2.275 × 10^4^	2.888 × 10^4^	3.644 × 10^4^
	Std	3.819 × 10^3^	9.210 × 10^3^	6.169 × 10^3^	1.537 × 10^4^	1.829 × 10^3^	3.146 × 10^3^	2.680 × 10^3^	9.035 × 10^3^
	Rank	1	5	6	7	2	3	4	8
F10	Mean	1.309 × 10^4^	2.392 × 10^4^	2.221 × 10^4^	2.477 × 10^4^	1.498 × 10^4^	1.373 × 10^4^	1.801 × 10^4^	2.001 × 10^4^
	Std	2.527 × 10^3^	8.570 × 10^3^	6.443 × 10^3^	8.391 × 10^3^	1.396 × 10^3^	1.385 × 10^3^	1.985 × 10^3^	2.483 × 10^3^
	Rank	1	7	6	8	3	2	4	5
F11	Mean	4.246 × 10^3^	4.117 × 10^2^	5.700 × 10^2^	5.684 × 10^3^	1.242 × 10^3^	1.368 × 10^3^	1.831 × 10^3^	7.394 × 10^3^
	Std	9.255 × 10^2^	1.035 × 10^2^	1.739 × 10^2^	8.984 × 10^3^	2.504 × 10^2^	1.912 × 10^2^	2.317 × 10^2^	2.505 × 10^3^
	Rank	6	1	2	7	3	4	5	8
F12	Mean	3.165 × 10^7^	1.149 × 10^7^	2.800 × 10^7^	6.203 × 10^7^	1.038 × 10^7^	4.020 × 10^7^	2.612 × 10^8^	6.965 × 10^8^
	Std	1.436 × 10^7^	6.168 × 10^6^	1.525 × 10^7^	3.738 × 10^7^	4.628 × 10^6^	2.197 × 10^7^	7.950 × 10^7^	2.299 × 10^8^
	Rank	4	2	3	6	1	5	7	8
F13	Mean	5.163 × 10^3^	1.207 × 10^4^	1.541 × 10^4^	2.274 × 10^4^	4.148 × 10^4^	7.740 × 10^4^	3.284 × 10^6^	1.321 × 10^5^
	Std	7.462 × 10^3^	1.279 × 10^4^	1.769 × 10^4^	2.354 × 10^4^	1.232 × 10^4^	3.422 × 10^4^	7.003 × 10^5^	2.216 × 10^5^
	Rank	1	2	3	4	5	6	8	7
F14	Mean	1.116 × 10^6^	3.697 × 10^5^	6.855 × 10^5^	1.511 × 10^6^	1.968 × 10^5^	2.596 × 10^5^	9.274 × 10^5^	1.598 × 10^6^
	Std	6.752 × 10^5^	1.997 × 10^5^	4.788 × 10^5^	7.230 × 10^5^	8.845 × 10^4^	1.440 × 10^5^	2.877 × 10^5^	8.335 × 10^5^
	Rank	6	3	4	7	1	2	5	8
F15	Mean	5.084 × 10^3^	6.587 × 10^3^	8.090 × 10^3^	9.253 × 10^3^	2.150 × 10^4^	5.470 × 10^4^	8.615 × 10^5^	9.355 × 10^4^
	Std	4.564 × 10^3^	9.642 × 10^3^	9.883 × 10^3^	1.285 × 10^4^	9.179 × 10^3^	2.241 × 10^4^	6.215 × 10^5^	1.370 × 10^5^
	Rank	1	2	3	4	5	6	8	7

**Table 2:** Experimental results of SLO, HCOA, ARBBPSO, ETBBPSO, AVOA, SSA, HHO, and WOA for F16–F29, with the average rank at the end of the table. Mean, Std, and Rand are dimensionless.

F	Data Type	SLO	HCOA	ARBBPSO	ETBBPSO	AVOA	SSA	HHO	WOA
F16	Mean	2.989 × 10^3^	5.178 × 10^3^	6.205 × 10^3^	6.884 × 10^3^	4.649 × 10^3^	4.039 × 10^3^	5.264 × 10^3^	7.823 × 10^3^
	Std	7.333 × 10^2^	1.352 × 10^3^	1.864 × 10^3^	2.684 × 10^3^	7.636 × 10^2^	7.771 × 10^2^	7.226 × 10^2^	1.916 × 10^3^
	Rank	1	4	6	7	3	2	5	8
F17	Mean	2.554 × 10^3^	4.029 × 10^3^	5.253 × 10^3^	4.680 × 10^3^	4.208 × 10^3^	3.489 × 10^3^	4.446 × 10^3^	5.236 × 10^3^
	Std	4.174 × 10^2^	6.142 × 10^2^	1.238 × 10^3^	9.030 × 10^2^	6.082 × 10^2^	5.701 × 10^2^	5.365 × 10^2^	9.383 × 10^2^
	Rank	1	3	8	6	4	2	5	7
F18	Mean	2.257 × 10^6^	1.233 × 10^6^	3.886 × 10^6^	7.196 × 10^6^	3.350 × 10^5^	3.891 × 10^5^	1.819 × 10^6^	2.133 × 10^6^
	Std	1.018 × 10^6^	5.233 × 10^5^	2.190 × 10^6^	3.634 × 10^6^	1.147 × 10^5^	1.620 × 10^5^	5.577 × 10^5^	1.031 × 10^6^
	Rank	6	3	7	8	1	2	4	5
F19	Mean	4.860 × 10^3^	1.349 × 10^4^	8.601 × 10^3^	1.622 × 10^4^	1.075 × 10^4^	1.773 × 10^6^	3.987 × 10^6^	1.301 × 10^7^
	Std	5.783 × 10^3^	1.635 × 10^4^	1.034 × 10^4^	1.298 × 10^4^	6.197 × 10^3^	5.689 × 10^5^	1.250 × 10^6^	5.502 × 10^6^
	Rank	1	4	2	5	3	6	7	8
F20	Mean	1.937 × 10^3^	3.128 × 10^3^	3.345 × 10^3^	3.908 × 10^3^	3.584 × 10^3^	2.859 × 10^3^	3.764 × 10^3^	4.061 × 10^3^
	Std	4.117 × 10^2^	6.372 × 10^2^	8.061 × 10^2^	1.220 × 10^3^	5.106 × 10^2^	4.240 × 10^2^	5.209 × 10^2^	5.735 × 10^2^
	Rank	1	3	4	7	5	2	6	8
F21	Mean	6.487 × 10^2^	9.988 × 10^2^	1.069 × 10^3^	1.155 × 10^3^	1.335 × 10^3^	8.691 × 10^2^	1.671 × 10^3^	1.788 × 10^3^
	Std	7.379 × 10^1^	1.244 × 10^2^	1.078 × 10^2^	2.041 × 10^2^	1.426 × 10^2^	1.178 × 10^2^	1.436 × 10^2^	2.245 × 10^2^
	Rank	1	3	4	5	6	2	7	8
F22	Mean	1.321 × 10^4^	2.175 × 10^4^	2.466 × 10^4^	2.733 × 10^4^	1.667 × 10^4^	1.643 × 10^4^	1.969 × 10^4^	2.258 × 10^4^
	Std	2.522 × 10^3^	8.547 × 10^3^	6.332 × 10^3^	8.054 × 10^3^	1.503 × 10^3^	1.476 × 10^3^	1.320 × 10^3^	2.605 × 10^3^
	Rank	1	5	7	8	3	2	4	6
F23	Mean	1.090 × 10^3^	1.217 × 10^3^	1.300 × 10^3^	1.289 × 10^3^	1.417 × 10^3^	1.153 × 10^3^	2.228 × 10^3^	2.458 × 10^3^
	Std	8.176 × 10^1^	9.620 × 10^1^	1.326 × 10^2^	1.227 × 10^2^	1.175 × 10^2^	1.038 × 10^2^	1.853 × 10^2^	1.574 × 10^2^
	Rank	1	3	5	4	6	2	7	8
F24	Mean	1.690 × 10^3^	1.763 × 10^3^	1.943 × 10^3^	1.913 × 10^3^	2.332 × 10^3^	1.522 × 10^3^	3.172 × 10^3^	3.522 × 10^3^
	Std	1.418 × 10^2^	1.506 × 10^2^	1.948 × 10^2^	1.774 × 10^2^	1.834 × 10^2^	1.259 × 10^2^	2.509 × 10^2^	3.620 × 10^2^
	Rank	2	3	5	4	6	1	7	8
F25	Mean	7.738 × 10^2^	7.393 × 10^2^	7.274 × 10^2^	7.641 × 10^2^	7.847 × 10^2^	7.847 × 10^2^	9.987 × 10^2^	1.125 × 10^3^
	Std	3.988 × 10^1^	5.786 × 10^1^	5.781 × 10^1^	7.991 × 10^1^	6.125 × 10^1^	6.040 × 10^1^	6.819 × 10^1^	8.146 × 10^1^
	Rank	4	2	1	3	6	5	7	8
F26	Mean	1.090 × 10^4^	1.282 × 10^4^	1.477 × 10^4^	1.470 × 10^4^	1.817 × 10^4^	8.298 × 10^3^	2.057 × 10^4^	2.849 × 10^4^
	Std	1.335 × 10^3^	2.037 × 10^3^	1.811 × 10^3^	1.915 × 10^3^	2.268 × 10^3^	4.136 × 10^3^	4.165 × 10^3^	2.442 × 10^3^
	Rank	2	3	5	4	6	1	7	8
F27	Mean	5.507 × 10^2^	5.000 × 10^2^	5.000 × 10^2^	5.000 × 10^2^	1.269 × 10^3^	9.294 × 10^2^	1.446 × 10^3^	2.396 × 10^3^
	Std	8.634 × 10^1^	5.695 × 10^−4^	3.074 × 10^−4^	4.462 × 10^−4^	2.559 × 10^2^	1.054 × 10^2^	2.901 × 10^2^	5.984 × 10^2^
	Rank	4	1	3	2	6	5	7	8
F28	Mean	5.045 × 10^2^	5.000 × 10^2^	5.000 × 10^2^	5.000 × 10^2^	5.624 × 10^2^	6.037 × 10^2^	7.649 × 10^2^	9.193 × 10^2^
	Std	1.565 × 10^1^	5.230 × 10^−4^	3.261 × 10^−4^	4.270 × 10^−4^	3.212 × 10^1^	3.818 × 10^1^	5.027 × 10^1^	5.448 × 10^1^
	Rank	4	1	3	2	5	6	7	8
F29	Mean	3.031 × 10^3^	3.757 × 10^3^	4.124 × 10^3^	4.261 × 10^3^	4.762 × 10^3^	5.259 × 10^3^	5.984 × 10^3^	1.038 × 10^4^
	Std	4.728 × 10^2^	8.213 × 10^2^	6.365 × 10^2^	8.008 × 10^2^	8.203 × 10^2^	6.019 × 10^2^	7.377 × 10^2^	1.730 × 10^3^
	Rank	1	2	3	4	5	6	7	8
Average Rank		2.4138	3.0690	4.4138	5.4138	3.8966	3.3793	6.1034	7.3103

**Table 3:** Details of datasets.

Dataset	Features	Instances	Classes
CL-SUB-111	11,340	111	3
Colon	2000	62	2
GLIOMA	4434	50	4
GLl-85	22,283	85	2
Lung	3312	203	5
Lymphoma	4026	96	9
Prostate-GE	5966	102	2
SMK-CAN-187	19,993	187	2

**Table 4:** Details of parameters.

Type	Parameter	Value
Optimization algorithm	Population	20
	Max iterations	100
	Dimension	feature number
KNN	Number of neighbors	5
K-Fold	K	10

**Table 5:** The fitness values results of SLO, HCOA, ARBBPSO, ETBBPSO, AVOA, SSA, HHO, and WOA. The average ranks are at the end of the table. Mean, Std, and Rand are dimensionless.

Dataset	Data Type	SLO	HCOA	ARBBPSO	ETBBPSO	AVOA	SSA	HHO	WOA
CL-SUB-111	Mean	2.1081 × 10^−1^	1.7710 × 10^−1^	2.0183 × 10^−1^	2.9062 × 10^−1^	2.0260 × 10^−1^	3.0041 × 10^−1^	2.4202 × 10^−1^	2.4210 × 10^−1^
	Std	1.8160 × 10^−2^	1.2566 × 10^−2^	1.4150 × 10^−2^	1.6813 × 10^−2^	2.8060 × 10^−2^	1.4559 × 10^−2^	4.0122 × 10^−2^	2.7449 × 10^−2^
	Rank	4	1	2	7	3	8	5	6
Colon	Mean	1.0348 × 10^−1^	1.6398 × 10^−1^	1.7162 × 10^−1^	2.3817 × 10^−1^	9.5990 × 10^−2^	2.2797 × 10^−1^	1.3011 × 10^−1^	1.4471 × 10^−1^
	Std	2.0939 × 10^−2^	1.4458 × 10^−2^	1.0693 × 10^−2^	1.6033 × 10^−2^	4.2088 × 10^−2^	1.1653 × 10^−2^	5.0435 × 10^−2^	3.3297 × 10^−2^
	Rank	2	5	6	8	1	7	3	4
GLIOMA	Mean	8.9906 × 10^−2^	1.4205 × 10^−1^	1.4318 × 10^−1^	2.1406 × 10^−1^	9.3696 × 10^−2^	2.1154 × 10^−1^	1.2347 × 10^−1^	1.2970 × 10^−1^
	Std	1.7388 × 10^−2^	1.7031 × 10^−2^	1.3279 × 10^−2^	1.7366 × 10^−2^	2.4294 × 10^−2^	1.8738 × 10^−2^	3.0976 × 10^−2^	3.1564 × 10^−2^
	Rank	1	5	6	8	2	7	3	4
GLl-85	Mean	4.7581 × 10^−2^	7.7917 × 10^−2^	8.4246 × 10^−2^	1.4512 × 10^−1^	6.8403 × 10^−2^	1.2756 × 10^−1^	8.7781 × 10^−2^	7.7577 × 10^−2^
	Std	7.8446 × 10^−3^	8.8051 × 10^−3^	1.0023 × 10^−2^	1.3105 × 10^−2^	1.6758 × 10^−2^	1.6558 × 10^−2^	2.4233 × 10^−2^	1.8331 × 10^−2^
	Rank	1	4	5	8	2	7	6	3
Lung	Mean	2.4853 × 10^−2^	2.8411 × 10^−2^	3.1052 × 10^−2^	4.4746 × 10^−2^	2.6576 × 10^−2^	4.2008 × 10^−2^	3.7438 × 10^−2^	3.6795 × 10^−2^
	Std	3.7882 × 10^−3^	3.2588 × 10^−3^	2.6659 × 10^−3^	3.6210 × 10^−3^	4.0332 × 10^−3^	2.6196 × 10^−3^	7.0013 × 10^−3^	6.7090 × 10^−3^
	Rank	1	3	4	8	2	7	6	5
Lymphoma	Mean	1.1358 × 10^−2^	2.3940 × 10^−2^	2.4120 × 10^−2^	3.9926 × 10^−2^	1.5733 × 10^−2^	3.7099 × 10^−2^	2.0977 × 10^−2^	2.1056 × 10^−2^
	Std	5.2336 × 10^−3^	3.0591 × 10^−3^	8.7428 × 10^−5^	5.6748 × 10^−3^	7.5777 × 10^−3^	6.6657 × 10^−3^	8.3754 × 10^−3^	6.9952 × 10^−3^
	Rank	1	5	6	8	2	7	3	4
Prostate-GE	Mean	8.4911 × 10^−2^	9.3291 × 10^−2^	9.9073 × 10^−2^	1.2449 × 10^−1^	8.9312 × 10^−2^	1.1990 × 10^−1^	1.0449 × 10^−1^	1.0918 × 10^−1^
	Std	8.0642 × 10^−3^	5.8976 × 10^−3^	7.6201 × 10^−3^	7.7733 × 10^−3^	1.4639 × 10^−2^	7.0833 × 10^−3^	1.6941 × 10^−2^	1.2125 × 10^−2^
	Rank	1	3	4	8	2	7	5	6
SMK-CAN-187	Mean	2.5062 × 10^−1^	2.7895 × 10^−1^	2.9507 × 10^−1^	3.3210 × 10^−1^	2.2902 × 10^−1^	3.2742 × 10^−1^	2.5349 × 10^−1^	2.7378 × 10^−1^
	Std	1.1201 × 10^−2^	7.4972 × 10^−3^	5.8515 × 10^−3^	7.6494 × 10^−3^	2.6161 × 10^−2^	7.0669 × 10^−3^	2.4594 × 10^−2^	1.4303 × 10^−2^
	Rank	2	5	6	8	1	7	3	4
Average rank		1.625	3.875	4.875	7.875	1.875	7.125	4.25	4.5

**Table 6:** The accuracy results of SLO, HCOA, ARBBPSO, ETBBPSO, AVOA, SSA, HHO, and WOA. The average ranks are at the end of the table. Mean, Std, and Rand are dimensionless.

Dataset	Data Type	SLO	HCOA	ARBBPSO	ETBBPSO	AVOA	SSA	HHO	WOA
CL-SUB-111	Mean	78.80%	82.59%	80.11%	65.74%	79.70%	70.16%	75.78%	75.64%
	Std	1.8182 × 10^−2^	1.2704 × 10^−2^	1.4299 × 10^−2^	3.0555 × 10^−2^	2.7779 × 10^−2^	1.4718 × 10^−2^	3.9040 × 10^−2^	2.7681 × 10^−2^
	Rank	4	1	2	8	3	7	5	6
Colon	Mean	89.56%	83.81%	83.11%	72.49%	90.35%	77.46%	86.91%	85.44%
	Std	2.1133 × 10^−2^	1.4665 × 10^−2^	1.0800 × 10^−2^	2.2376 × 10^−2^	4.2169 × 10^−2^	1.1752 × 10^−2^	5.0375 × 10^−2^	3.3235 × 10^−2^
	Rank	2	5	6	8	1	7	3	4
GLIOMA	Mean	90.94%	86.06%	86.00%	74.31%	90.56%	79.13%	87.56%	86.94%
	Std	1.7586 × 10^−2^	1.7215 × 10^−2^	1.3440 × 10^−2^	2.2781 × 10^−2^	2.4487 × 10^−2^	1.8965 × 10^−2^	3.1206 × 10^−2^	3.1718 × 10^−2^
	Rank	1	5	6	8	2	7	3	4
GLl-85	Mean	95.28%	92.60%	91.98%	81.08%	93.16%	87.62%	91.24%	92.27%
	Std	7.9472 × 10^−3^	8.9193 × 10^−3^	1.0146 × 10^−2^	2.4462 × 10^−2^	1.6804 × 10^−2^	1.6730 × 10^−2^	2.3797 × 10^−2^	1.8390 × 10^−2^
	Rank	1	3	5	8	2	7	6	4
Lung	Mean	97.59%	97.53%	97.32%	94.64%	97.45%	96.25%	96.46%	96.51%
	Std	3.8822 × 10^−3^	3.3417 × 10^−3^	2.7004 × 10^−3^	6.9742 × 10^−3^	3.7946 × 10^−3^	2.6715 × 10^−3^	6.2936 × 10^−3^	6.0245 × 10^−3^
	Rank	1	2	4	8	3	7	6	5
Lymphoma	Mean	98.90%	97.97%	98.00%	95.19%	98.47%	96.74%	97.99%	97.96%
	Std	5.2977 × 10^−3^	3.0946 × 10^−3^	2.2560 × 10^−16^	8.2254 × 10^−3^	7.6134 × 10^−3^	6.7524 × 10^−3^	8.1276 × 10^−3^	6.9510 × 10^−3^
	Rank	1	5	3	8	2	7	4	6
Prostate-GE	Mean	91.51%	91.00%	90.47%	85.11%	91.12%	88.39%	89.64%	89.20%
	Std	7.9784 × 10^−3^	5.9836 × 10^−3^	7.7578 × 10^−3^	1.2511 × 10^−2^	1.4291 × 10^−2^	7.1694 × 10^−3^	1.6283 × 10^−2^	1.1145 × 10^−2^
	Rank	1	3	4	8	2	7	5	6
SMK-CAN-187	Mean	74.70%	72.31%	70.70%	64.66%	76.89%	67.43%	74.41%	72.39%
	Std	1.1304 × 10^−2^	7.5692 × 10^−3^	5.8964 × 10^−3^	1.2856 × 10^−2^	2.6312 × 10^−2^	7.1359 × 10^−3^	2.4689 × 10^−2^	1.4238 × 10^−2^
	Rank	2	5	6	8	1	7	3	4
Avg		1.625	3.625	4.5	8	2	7	4.375	4.375

**Table 7:** The feature selection number result of SLO, HCOA, ARBBPSO, ETBBPSO, AVOA, SSA, HHO, and WOA. Mean, Std, and Rand are dimensionless.

Dataset	Data Type	SLO	HCOA	ARBBPSO	ETBBPSO	AVOA	SSA	HHO	WOA
CL-SUB-111	Mean	1056.91	5377.75	5597.09	5664.56	1816.91	5646.84	2555.03	1025.53
	Std	9.2987 × 10^2^	7.1767 × 10^1^	6.1767 × 10^1^	5.8900 × 10^1^	1.4839 × 10^3^	4.7480 × 10^1^	2.2937 × 10^3^	9.6652 × 10^2^
Colon	Mean	27.28	739.56	881.56	989.69	90.50	971.06	108.44	110.72
	Std	2.2795 × 10^1^	2.6521 × 10^1^	3.1963 × 10^1^	2.0059 × 10^1^	8.9478 × 10^1^	2.0299 × 10^1^	1.4874 × 10^2^	1.1967 × 10^2^
GLIOMA	Mean	83.06	1805.06	2029.69	2206.22	117.41	2164.84	150.91	170.91
	Std	4.7916 × 10^1^	3.9152 × 10^1^	4.3052 × 10^1^	3.5398 × 10^1^	8.0773 × 10^1^	4.3664 × 10^1^	1.1466 × 10^2^	1.8859 × 10^2^
GLl-85	Mean	1948.34	10,374.06	10,881.06	11,159.28	1544.16	11,079.72	2289.25	2242.34
	Std	8.8467 × 10^2^	1.0361 × 10^2^	9.5213 × 10^1^	6.7427 × 10^1^	9.6098 × 10^2^	7.0375 × 10^1^	2.2063 × 10^3^	1.4334 × 10^3^
Lung	Mean	314.66	1312.50	1511.41	1655.84	453.63	1631.94	806.28	737.25
	Std	1.6051 × 10^2^	3.4252 × 10^1^	3.7461 × 10^1^	2.6910 × 10^1^	1.8559 × 10^2^	3.0064 × 10^1^	4.7837 × 10^2^	5.1888 × 10^2^
Lymphoma	Mean	185.56	1542.25	1739.34	2008.50	230.84	1954.66	432.28	353.38
	Std	6.3346 × 10^1^	3.8491 × 10^1^	3.5199 × 10^1^	3.1842 × 10^1^	1.1206 × 10^2^	3.6470 × 10^1^	3.4403 × 10^2^	2.0329 × 10^2^
Prostate-GE	Mean	504.63	2517.03	2795.44	2976.78	848.19	2935.72	1145.34	1360.69
	Std	2.8526 × 10^2^	4.2483 × 10^1^	6.2382 × 10^1^	3.1119 × 10^1^	4.5602 × 10^2^	3.8881 × 10^1^	8.8651 × 10^2^	1.0756 × 10^3^
SMK-CAN-187	Mean	274.56	9676.47	9931.63	9984.22	394.53	9964.28	341.09	865.69
	Std	2.1574 × 10^2^	1.0269 × 10^2^	7.4833 × 10^1^	6.4971 × 10^1^	4.1812 × 10^2^	6.7734 × 10^1^	5.8832 × 10^2^	7.7296 × 10^2^

**Table 8:** The fitness values results of SLO, HCOA, ARBBPSO, ETBBPSO, AVOA, SSA, HHO, and WOA. The average ranks are at the end of the table. Mean, Std, and Rank are dimensionless.

Dataset	Data Type	SLO	HCOA	ARBBPSO	ETBBPSO	AVOA	SSA	HHO	WOA
CL-SUB-111	Mean	2.5631 × 10^−1^	2.7450 × 10^−1^	3.0892 × 10^−1^	3.7347 × 10^−1^	2.1584 × 10^−1^	3.5981 × 10^−1^	2.2642 × 10^−1^	2.6891 × 10^−1^
	Std	3.7738 × 10^−2^	1.4196 × 10^−2^	1.1081 × 10^−2^	1.3198 × 10^−2^	3.7745 × 10^−2^	1.9297 × 10^−2^	4.6073 × 10^−2^	4.5172 × 10^−2^
	Rank	3	5	6	8	1	7	2	4
Colon	Mean	1.0026 × 10^−1^	1.1115 × 10^−1^	1.2875 × 10^−1^	1.7737 × 10^−1^	9.3892 × 10^−2^	1.7139 × 10^−1^	1.1981 × 10^−1^	1.2513 × 10^−1^
	Std	1.0139 × 10^−2^	1.7802 × 10^−2^	1.0810 × 10^−2^	1.1407 × 10^−2^	2.1744 × 10^−2^	1.2137 × 10^−2^	3.2057 × 10^−2^	2.4078 × 10^−2^
	Rank	2	3	6	8	1	7	4	5
GLIOMA	Mean	7.9639 × 10^−2^	8.5780 × 10^−2^	9.3771 × 10^−2^	1.3535 × 10^−1^	7.3240 × 10^−2^	1.2746 × 10^−1^	8.5718 × 10^−2^	9.8278 × 10^−2^
	Std	1.0220 × 10^−2^	8.0884 × 10^−3^	7.8341 × 10^−3^	1.1425 × 10^−2^	1.6098 × 10^−2^	1.1117 × 10^−2^	2.3884 × 10^−2^	1.3943 × 10^−2^
	Rank	2	4	5	8	1	7	3	6
GLl-85	Mean	1.0811 × 10^−1^	1.3094 × 10^−1^	1.3245 × 10^−1^	1.4678 × 10^−1^	1.0890 × 10^−1^	1.4342 × 10^−1^	1.3408 × 10^−1^	1.2296 × 10^−1^
	Std	9.9462 × 10^−3^	9.8691 × 10^−3^	9.9462 × 10^−3^	8.8298 × 10^−3^	1.4809 × 10^−2^	4.7108 × 10^−3^	1.1947 × 10^−2^	1.3159 × 10^−2^
	Rank	1	4	5	8	2	7	6	3
Lung	Mean	2.0455 × 10^−2^	2.3487 × 10^−2^	2.2994 × 10^−2^	3.7960 × 10^−2^	2.3693 × 10^−2^	3.5112 × 10^−2^	3.3044 × 10^−2^	3.2522 × 10^−2^
	Std	3.1725 × 10^−3^	3.8029 × 10^−3^	2.8117 × 10^−3^	4.8553 × 10^−3^	4.8199 × 10^−3^	3.6576 × 10^−3^	4.6249 × 10^−3^	4.3614 × 10^−3^
	Rank	1	3	2	8	4	7	6	5
Lymphoma	Mean	4.1896 × 10^−2^	5.0324 × 10^−2^	5.1410 × 10^−2^	6.3265 × 10^−2^	4.4477 × 10^−2^	6.1090 × 10^−2^	5.6761 × 10^−2^	5.3448 × 10^−2^
	Std	3.6803 × 10^−3^	4.5993 × 10^−3^	5.5571 × 10^−3^	5.2229 × 10^−3^	4.9059 × 10^−3^	6.1363 × 10^−3^	7.7438 × 10^−3^	8.0177 × 10^−3^
	Rank	1	3	4	8	2	7	6	5
Prostate-GE	Mean	7.0119 × 10^−2^	8.1605 × 10^−2^	8.2322 × 10^−2^	1.0428 × 10^−1^	7.1268 × 10^−2^	9.6563 × 10^−2^	8.4776 × 10^−2^	8.3153 × 10^−2^
	Std	7.7614 × 10^−3^	1.7373 × 10^−4^	1.6538 × 10^−3^	9.1685 × 10^−3^	1.3136 × 10^−2^	8.9399 × 10^−3^	1.6637 × 10^−2^	1.1965 × 10^−2^
	Rank	1	3	4	8	2	7	6	5
SMK-CAN-187	Mean	2.2291 × 10^−1^	2.6034 × 10^−1^	2.8003 × 10^−1^	3.1572 × 10^−1^	2.3185 × 10^−1^	3.0834 × 10^−1^	2.4881 × 10^−1^	2.5840 × 10^−1^
	Std	1.6706 × 10^−2^	8.2753 × 10^−3^	7.9118 × 10^−3^	8.4489 × 10^−3^	1.8346 × 10^−2^	7.0899 × 10^−3^	2.0267 × 10^−2^	1.5261 × 10^−2^
	Rank	1	5	6	8	2	7	3	4
Avg		1.5	3.75	4.75	8	1.875	7	4.5	4.625

**Table 9:** The accuracy results of SLO, HCOA, ARBBPSO, ETBBPSO, AVOA, SSA, HHO, and WOA. Mean, Std, and Rank are dimensionless.

Dataset	Data Type	SLO	HCOA	ARBBPSO	ETBBPSO	AVOA	SSA	HHO	WOA
CL-SUB-111	Mean	74.14%	72.76%	69.30%	57.56%	78.21%	64.16%	77.15%	72.88%
	Std	3.7884 × 10^−2^	1.4338 × 10^−2^	1.1191 × 10^−2^	2.4561 × 10^−2^	3.8078 × 10^−2^	1.9491 × 10^−2^	4.6096 × 10^−2^	4.5079 × 10^−2^
	Rank	3	5	6	8	1	7	2	4
Colon	Mean	89.92%	89.16%	87.45%	78.43%	90.60%	83.18%	88.02%	87.45%
	Std	1.0134 × 10^−2^	1.8101 × 10^−2^	1.0901 × 10^−2^	2.3177 × 10^−2^	2.1736 × 10^−2^	1.2288 × 10^−2^	3.1402 × 10^−2^	2.3957 × 10^−2^
	Rank	2	3	6	8	1	7	4	5
GLIOMA	Mean	92.00%	91.81%	91.03%	82.57%	92.70%	87.63%	91.45%	90.21%
	Std	1.0208 × 10^−2^	8.1996 × 10^−3^	7.9146 × 10^−3^	2.4621 × 10^−2^	1.5935 × 10^−2^	1.1241 × 10^−2^	2.3092 × 10^−2^	1.3763 × 10^−2^
	Rank	2	3	5	8	1	7	4	6
GLl-85	Mean	89.11%	87.17%	87.06%	83.06%	89.06%	86.00%	86.61%	87.67%
	Std	1.0079 × 10^−2^	1.0000 × 10^−2^	1.0126 × 10^−2^	2.1639 × 10^−2^	1.4725 × 10^−2^	4.7809 × 10^−3^	1.1533 × 10^−2^	1.3093 × 10^−2^
	Rank	1	4	5	8	2	7	6	3
Lung	Mean	98.35%	97.76%	98.15%	95.40%	97.78%	96.95%	96.94%	96.96%
	Std	3.2897 × 10^−3^	3.9348 × 10^−3^	2.8562 × 10^−3^	6.2770 × 10^−3^	4.8616 × 10^−3^	3.7213 × 10^−3^	4.0458 × 10^−3^	4.3547 × 10^−3^
	Rank	1	4	2	8	3	6	7	5
Lymphoma	Mean	95.83%	95.31%	95.24%	92.69%	95.58%	94.32%	94.45%	94.74%
	Std	3.7796 × 10^−3^	4.6718 × 10^−3^	5.6893 × 10^−3^	1.0384 × 10^−2^	5.0000 × 10^−3^	6.1994 × 10^−3^	7.3241 × 10^−3^	7.8304 × 10^−3^
	Rank	1	3	4	8	2	7	6	5
Prostate-GE	Mean	92.99%	92.18%	92.15%	87.04%	92.91%	90.74%	91.62%	91.73%
	Std	7.6960 × 10^−3^	1.5152 × 10^−4^	1.6667 × 10^−3^	1.2196 × 10^−2^	1.2676 × 10^−2^	9.0410 × 10^−3^	1.5477 × 10^−2^	1.1477 × 10^−2^
	Rank	1	3	4	8	2	7	6	5
SMK-CAN-187	Mean	77.50%	74.19%	72.21%	65.94%	76.60%	69.36%	74.88%	73.93%
	Std	1.6799 × 10^−2^	8.3617 × 10^−3^	7.9923 × 10^−3^	8.9688 × 10^−3^	1.8417 × 10^−2^	7.1520 × 10^−3^	2.0344 × 10^−2^	1.5116 × 10^−2^
	Rank	1	4	6	8	2	7	3	5
Avg		1.5	3.625	4.75	8	1.75	6.875	4.75	4.75

**Table 10:** The feature selection numerical results of SLO, HCOA, ARBBPSO, ETBBPSO, AVOA, SSA, HHO, and WOA. Mean, Std, and Rank are dimensionless.

Dataset	Data Type	SLO	HCOA	ARBBPSO	ETBBPSO	AVOA	SSA	HHO	WOA
CL-SUB-111	Mean	348.19	5478.44	5623.00	5660.33	150.92	5646.39	287.97	507.86
	Std	5.9346 × 10^2^	9.2933 × 10^1^	5.8130 × 10^1^	6.0862 × 10^1^	1.2166 × 10^2^	5.7050 × 10^1^	9.7598 × 10^2^	1.2222 × 10^3^
Colon	Mean	95.42	766.50	907.97	994.17	170.00	977.64	234.22	183.39
	Std	7.1993 × 10^1^	3.5813 × 10^1^	3.6799 × 10^1^	2.5303 × 10^1^	1.2397 × 10^2^	2.5776 × 10^1^	2.6281 × 10^2^	1.6830 × 10^2^
GLIOMA	Mean	1029.28	10,542.78	10,986.86	11,121.64	2260.33	11,081.25	2404.47	2986.44
	Std	7.1368 × 10^2^	1.6054 × 10^2^	1.0474 × 10^2^	6.8322 × 10^1^	2.0342 × 10^3^	7.4274 × 10^1^	3.1728 × 10^3^	2.7974 × 10^3^
GLl-85	Mean	135.94	1722.67	1905.81	2201.97	245.97	2136.06	676.92	382.14
	Std	7.4089 × 10^1^	2.9944 × 10^1^	4.6727 × 10^1^	4.0290 × 10^1^	1.7948 × 10^2^	3.4238 × 10^1^	5.3719 × 10^2^	3.9691 × 10^2^
Lung	Mean	1364.06	438.19	1545.94	1655.33	558.58	1632.00	925.22	795.72
	Std	4.5686 × 10^1^	1.8381 × 10^2^	3.1863 × 10^1^	2.7088 × 10^1^	2.4242 × 10^2^	2.7843 × 10^1^	5.2599 × 10^2^	3.6730 × 10^2^
Lymphoma	Mean	260.06	1549.61	1740.89	2009.72	302.67	1959.86	746.00	543.83
	Std	1.2993 × 10^2^	2.9897 × 10^1^	5.6864 × 10^1^	3.3081 × 10^1^	1.5301 × 10^2^	3.8429 × 10^1^	4.6672 × 10^2^	3.6865 × 10^2^
Prostate-GE	Mean	414.19	2494.06	2772.39	2972.14	622.28	2945.94	1089.31	792.31
	Std	4.1223 × 10^2^	4.1517 × 10^1^	4.7354 × 10^1^	4.1481 × 10^1^	4.6875 × 10^2^	3.5731 × 10^1^	1.1068 × 10^3^	6.5756 × 10^2^
SMK-CAN-187	Mean	297.00	9730.39	9892.61	9989.28	367.47	9971.64	278.47	613.50
	Std	4.7930 × 10^2^	1.0029 × 10^2^	6.0723 × 10^1^	6.2317 × 10^1^	4.5571 × 10^2^	8.4956 × 10^1^	4.3765 × 10^2^	1.2489 × 10^3^

## Data Availability

The data presented in this study are available on request from the authors.

## Abbreviations

The following abbreviations are used in this manuscript:

BOA	Butterfly Optimization Algorithm
POSCO	Pelican Optimization Single Candidate Optimizer
GAPSO	Greedy-Strategy-Based Adaptive Particle Swarm Optimization
HSCA	Hybrid Sine Cosine Algorithm
WOA	Whale Optimization Algorithm
ELO	Enhanced Lemurs Optimization
AVOA	African Vultures Optimization Algorithm
ARO	Artificial Rabbit Optimization
HCOA	Hermit Crab Optimization Algorithm
GOA	Gazelle Optimization Algorithm
SO	Snake Optimizer
HHO	Harris Hawks Optimizer
SSA	Sparrow Search Algorithm
TDO	Tasmanian Devil Optimization
CHO	Cat Hunting Optimization
HBA	Honey Badger Algorithm
HOA	Horse herd Optimization Algorithm
SLO	Snow Leopard Optimization
BC	behavioral control
ME	mean error
Std	standard deviation
KNN	K Nearest Neighbor
